# Novel multidisciplinary approach to monitor and treat fetuses with gastroschisis using the Svetliza Reducibility Index and the EXIT-like procedure

**DOI:** 10.1590/S1679-45082017AO3979

**Published:** 2017

**Authors:** Gustavo Henrique de Oliveira, Javier Svetliza, Denise Cristina Mós Vaz-Oliani, Humberto Liedtke, Antonio Helio Oliani, Denise Araujo Lapa Pedreira

**Affiliations:** 1 Faculdade de Medicina de São José do Rio Preto, São José do Rio Preto, SP, Brazil.; 2 Universidade de São Paulo, São Paulo, SP, Brazil.

**Keywords:** Gastroschisis/surgery, Abdominal wall/abnormalities, Fetal therapies, Fetal diagnosis, Fetus/surgery, Gastrosquise/cirurgia, Parede abdominal/anormalidades, Terapias fetais, Diagnóstico fetal, Feto/cirurgia

## Abstract

**Objective:**

To describe our initial experience with a novel approach to follow-up and treat gastroschisis in “zero minute” using the EXITlike procedure.

**Methods:**

Eleven fetuses with prenatal diagnosis of gastroschisis were evaluated. The Svetliza Reductibility Index was used to prospectively evaluate five cases, and six cases were used as historical controls. The Svetliza Reductibility Index consisted in dividing the real abdominal wall defect diameter by the larger intestinal loop to be fitted in such space. The EXIT-like procedure consists in planned cesarean section, fetal analgesia and return of the herniated viscera to the abdominal cavity before the baby can fill the intestines with air. No general anesthesia or uterine relaxation is needed. Exteriorized viscera reduction is performed while umbilical cord circulation is maintained.

**Results:**

Four of the five cases were performed with the EXIT-like procedure. Successful complete closure was achieved in three infants. The other cases were planned deliveries at term and treated by construction of a Silo. The average time to return the viscera in EXIT-like Group was 5.0 minutes, and, in all cases, oximetry was maintained within normal ranges. In the perinatal period, there were significant statistical differences in ventilation days required (p = 0.0169), duration of parenteral nutrition (p=0.0104) and duration of enteral feed (p=0.0294).

**Conclusion:**

The Svetliza Reductibility Index and EXIT-like procedure could be new options to follow and treat gastroschisis, with significantly improved neonatal outcome in our unit. Further randomized studies are needed to evaluate this novel approach.

## RESUMO

**Objetivo:**

Descrever a experiência inicial com a nova técnica cirúrgica *EXIT-like* para acompanhamento e correção de gastrosquise no “minuto zero”.

**Métodos:**

Foram avaliados onze fetos com diagnóstico pré-natal de gastrosquise. O *Svetliza Reducibility Index* foi usado prospectivamente para avaliar cinco casos, e seis foram utilizados como controles. O índice foi calculado dividindo-se o diâmetro do defeito da parede abdominal pela maior alça intestinal que coubesse neste espaço. O procedimento *EXIT-like* compreendeu cesárea programada, analgesia fetal e redução da víscera herniada para a cavidade abdominal, antes da deglutição de ar pelo recém- nascido. Não são necessários anestesia geral e nem relaxamento uterino. A redução da víscera exteriorizada é realizada enquanto se mantém a circulação do cordão umbilical.

**Resultados:**

Quatro casos, dos cinco, foram submetidos ao *EXIT-like.* A correção foi completa em três casos. Os demais casos foram partos a termo planejados, e a correção do defeito foi feita com Silo. O tempo médio de redução da víscera foi de 5,0 minutos no grupo submetido ao procedimento *EXITlike,* e a oximetria foi mantida dentro dos valores de variação normal em todos os casos. No período perinatal, foram observadas diferenças estatisticamente significativas no tempo de ventilação mecânica (p=0,0169), duração da nutrição parenteral (p = 0,0104) e da nutrição enteral (p=0,0294).

**Conclusão:**

O *Svetliza Reducibility Index* e o procedimento *EXIT-like* podem ser novas opções para acompanhar e tratar gastroquise, com desfecho neonatal significativamente melhor em nossa unidade. Novos estudos randomizados são necessários para avaliar esta nova abordagem.

## INTRODUCTION

Gastroschisis is characterized by a defect of closure of the anterior abdominal wall, which is associated to the extrusion of abdominal organs, mainly bowel, stomach, bladder and liver. It is a low morbidity and mortality malformation; however, it is becoming more frequent with an estimate of 1:2,000-3,000 born alive.^(^ ^1^ ^,^ ^2^ ^)^

Although gastroschisis is a relatively benign malformation, there is no consensus about the best algorithm for its treatment. There has been a lot of controversy about gestational age at delivery, as well as the best surgical technique to be performed.^(^ ^1^ ^,^ ^3^ ^-^ ^5^ ^)^ Some studies suggest that elective earlier delivery anticipation may reduce intestinal damage and enhance neonatal outcome.^(^ ^1^ ^,^ ^6^ ^-^ ^11^ ^)^

To date, gastroschisis treatment is recommended to be performed as early as possible after birth, to avoid herniated viscera evaporation and exudation that may lead to dehydration, hypothermia and neonatal infection.^(^ ^1^ ^)^ The main techniques currently used are the primary closure of the defect with complete reduction of herniated contents, or gradual reduction during the first days of life by using a preformed or a customized Silo.^(^ ^12^ ^-^ ^17^ ^)^

Since 2005, a multidisciplinary group in Argentina has started to use a new approach combining intuitive ultrasonographic parameters and immediate “zero minute” surgeries to treat the disease, called EXITlike (ex utero intrapartum treatment) procedure. Their goal was to predict the largest intestinal loop diameter that could allow a primary reduction of the exteriorized viscera back to the abdominal cavity immediately at birth, before air started to distend the bowel once the neonate started to breathe. A planned cesarean-section allowed the pediatric surgeon to operate while the umbilical cord circulation was maintained. However, unlike the classic EXIT, no maternal general anesthesia or uterine relaxation was required. Then the umbilical cord was clamped and sectioned, and the neonate was moved to the neonatal isolette, where the abdominal wall defect closure could be completed.^(^ ^18^ ^)^

## OBJECTIVE

To present an initial experience with the proposal of a “zero minute” surgical correction through EXIT-like technique to treat gastroschisis.

## METHODS

All cases of prenatal diagnosis of gastroschisis in our region are evaluated at the *Hospital da Criança e Maternidade* (HCM) of the *Fundação Faculdade Regional de Medicina de São José do Rio Preto* which is a tertiary referral center for fetal malformations in the northeast of São Paulo, which includes fetal medicine experts, anesthesiologists, pediatric surgeons, obstetricians, neonatologists and other professionals. The study was approved by the Research Ethics Committe, under the resolution 1.864.004, CAAE: 62913216.4.0000.5415. The mothers signed the Informed Consent Form, and authorized data collection and publication of results.

**Figure 1:**
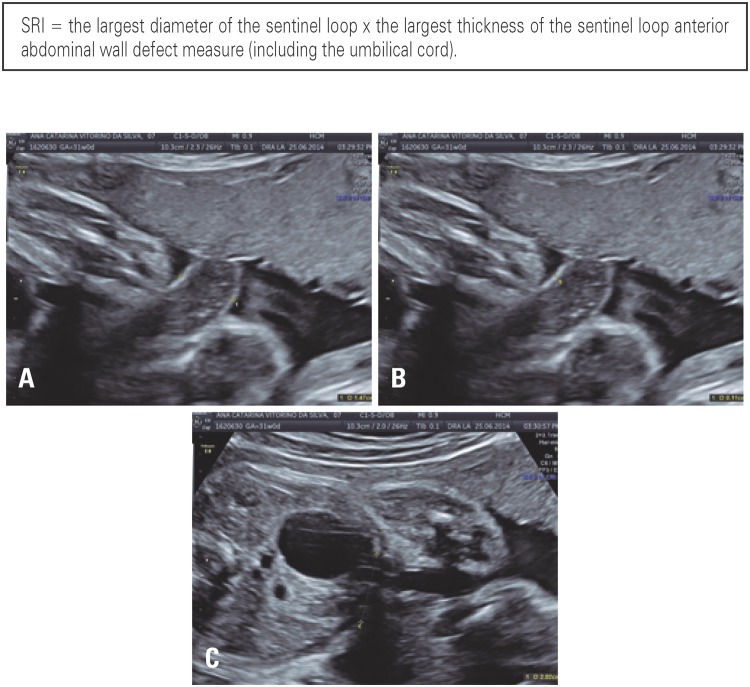
Ultrasonographic images to calculate the Svetliza Reducibility Index. (A) The largest diameter of the sentinel loop; (B) the largest thickness of the sentinel loop; (C) anterior abdominal wall defect measure (including the umbilical cord)

A change in the gastroschisis treatment protocol was proposed, and five cases were submitted to prospective evaluation for elective scheduled delivery between 35 and 37 gestational weeks. From 28 weeks of gestation on, the pregnant women underwent weekly ultrasound follow-up. The longitudinal follow-up of the reducibility index was useful to decide about the best moment for delivery. This index, proposed by Svetliza, Svetliza Reducibility Index (SRI) is calculated by multiplying the largest diameter by the largest thickness of the sentinel loop - defined as the bowel and extraabdominal loop with the largest dilatation. This value is divided by the largest measure of the anterior abdominal wall defect (including the umbilical cord) (Figure 1).

The interpretation of SRI values was carried out as follows: if less than or equal to 1.5, EXIT-like correction was considered applicable and probable; if greater than 1.5 and less than 2.5, EXIT-like correction was considered applicable and possible; and if greater than or equal to 2.5, EXIT-like correction was considered unlikely to occur.

The aim of evaluating the index prospectively was to ensure the opportunity of a “zero minute” surgery, that is, if SRI was less than 1.5, delivery was scheduled between 36 and 37 weeks, and if the index was progressively increasing before it exceeded 2.5, delivery was scheduled for 34 weeks of gestation or later.

The EXIT-like technique was planned in cases when SRI was lower than 2.5, therefore favorable to primary reduction at “zero minute”. This procedure is characterized by a complete reintroduction of bowel loops in fetal abdomen with the support of fetal- placental circulation, that is, before umbilical cord clamping. It differs from the classic EXIT technique, since general anesthesia or uterine relaxation drugs are not required.^(^ ^18^ ^)^

For the EXIT-like technique, the operating room was prepared as usual for a cesarean section, plus an accessory table with the material necessary for gastroschisis repair, including atraumatic tweezers to safely manipulate bowel loops, in addition to a heated bassinet for the newborns to be used after umbilical cord clamping. Also, there was another operating room prepared if general anesthesia is needed for the newborn.

The maternal anesthesia was performed by subarachnoid blockage with 10 to 20mg bupivacaine, depending on the weight and height of the patient, 100*μ*g of morphine, as well as oxygen mask as ventilatory support. As for the neonate analgesia, remifentanil at a dose of 0.07 to 0.09mg/kg/minute was administered in an infusion pump in maternal circulation, beginning at least 15 minutes before fetal extraction and maintained until umbilical cord clamp. The newborn was also offered a sucrose-dipped pacifier (5g of sucrose diluted in 100mL of water). Temperature was kept and the bassinet was less than 1m away from the operating table.

**Figure 2:**
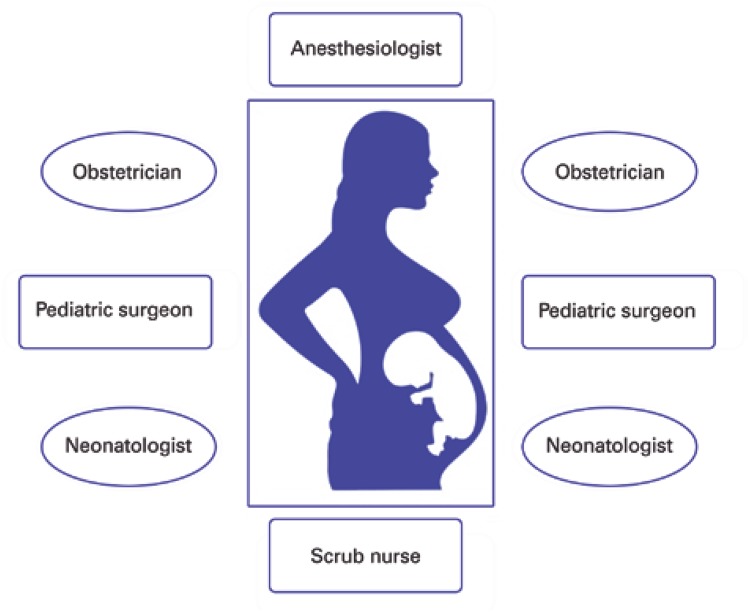
Schematic diagram of the surgical team positioning

**Figure 3:**
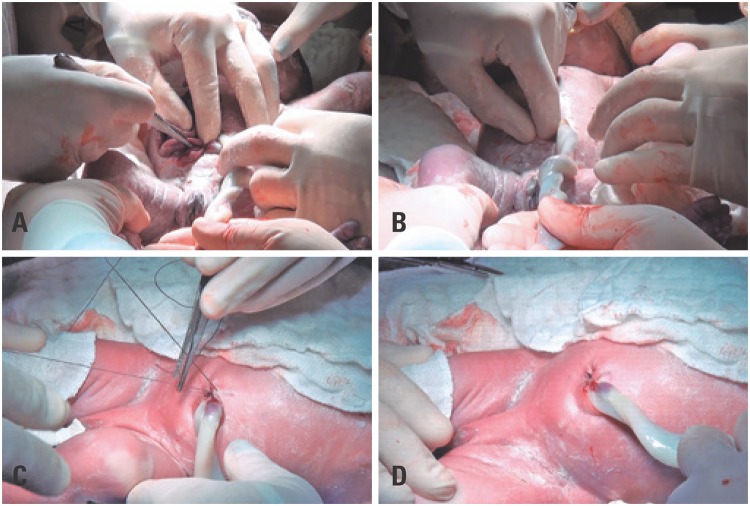
EXIT-like surgical steps. (A and B) Gastroschisis reduction with umbilical circulation; (C and D) abdominal wall closure

The surgical team included two anesthesiologists, two obstetricians, two pediatric surgeons, two neonatologists and a scrub nurse. Their positions in the operating room are shown in figure 2. After a smooth extraction of the fetus, it was placed in a supine position on maternal thighs, paying close attention not to cause excessive traction on the umbilical cord. A neonatologist offered the sucrose-dipped pacifier in order to prevent crying, while the other placed a pulse oximeter for monitoring. An obstetrician was in charge of protecting the surgical area of the cesarean, while the other observed umbilical cord pulsation by uninterrupted palpitation. Both pediatric surgeons started abdominal organ reduction, from the more dilated bowel loops to the less dilated ones until their total reduction. After umbilical cord clamp, the newborn was taken to a heated bassinet for a local anesthetic blockage of the abdominal wall with a 2mg/kg dosage of bupivacaine, and suture in a single layer with 3.0 absorbable thread (Figure 3).

Post-operatory parameters analyzed in all cases were: duration of mechanical ventilation, duration of exclusive and mixed parenteral nutrition, total enteral nutrition, sepsis occurrence, need for reintervention and length of hospital stay. These data were compared to the results in which a polyvinyl chloride (PVC) Silo placement was performed.

Six consecutive cases treated with Silo were chosen as controls and were retrospectively evaluated. Before the EXIT-like protocol, cases were managed as watchful waiting until term gestation. The delayed closure by Silo placement was always the first choice and primary repair was considered as an alternative in cases with low herniated content and easy reduction. The change of clinical approach was approved by the fetal medicine, obstetrics, neonatology and pediatric surgery teams. The medical ethics committee of the organization was consulted for compassionate procedure.

Data analysis was based on descriptive statistics calculations, frequency tables and nonparametric Mann- Whitney test; the statistical significance level was set at 5%.

## RESULTS

In the five cases prospectively evaluated, SRI was less than 2.5 in four cases submitted to the EXIT-like technique. In the case in which SRI was not favorable (greater than 2.5) and in the six control cases, gastroschisis correction was carried out by classic delayed closure using a customized Silo. The demographic characteristics of the cases are shown in table 1.

**Table 1:** Demographic characteristics

Characteristics		EXIT-like Group n (%) or mean (range) n=4	Silo Group n (%) or mean (range) n=7
Maternal age at delivery, years		21.5 (15-33)	20.14 (14-29)
Parity			
	Primípara	3 (75)	5 (71.73)
	Multipara	1 (25)	2 (28.57)
Fetal sex			
	Male	2 (50)	4 (57.14)
	Female	2 (50)	3 (42.86)
Gestational age at delivery, weeks		35.61 (35.00-36.43)	37.08 (35.29-38.43)
Birth weight, g		2,189.25 (1,556-2,975)	2,420 (1,700-3,480)
Time for herniated viscera reduction		5.0 minutes (4.0-5.8)	11.6 days (6.0-18.0)

Maternal age varied from 14 to 33 years (half of them under 19 years). The same multidisciplinary team was involved in the discussion, planning and surgery in all cases. In three cases, a complete bowel loops reduction was successfully performed with umbilical circulation and, in one case, after a reduction of approximately 50% of the herniated content, a superficial laceration of the small bowel loop was identified probably secondary to the manipulation; therefore the EXIT-like surgery was abandoned and a Silo was placed in the adjacent (prepared in advance) operating room. The average time to reduce bowel loops to the interior of the abdominal cavity was 5.0 minutes (4.0 to 5.8 minutes). In all cases, during bowel loops reduction procedure and abdominal wall closure, the newborn remained breathing spontaneously and with appropriate levels of oxygen saturation. In one case, approximately 30 minutes after abdominal wall closure, the newborn developed respiratory discomfort and oxygen desaturation, so mechanical ventilation was carried out; extubation occurred on the third post-operatory day. In the case with laceration of the small bowel loop, the newborn underwent general anestesia and was extubated right after the procedure. Post-operatory results of the four EXIT-like cases are individually demonstrated in table 2. The results and statistical differences concerning the EXIT-like procedure and the traditional Silo closure are shown in table 3.

**Table 2:** Cases submitted to the EXIT-like technique

Case	Gestational age at birth (weeks)	Weight at birth (g)	Mechanical ventilation (days)	Parenteral nutrition (days)	Mixed nutrition (days)	Enteral nutrition (days)	Sepsis	Reintervation	Hospital stay (days)
1	35.0	2,080	0	15	9	24	Yes	No	34
2*	36.71	2,975	1	18	7	25	No	No	33
3	35.29	2,146	3	14	22	36	No	No	43
4	35.71	1,556	0	14	12	26	No	No	46

* Case in which superficial laceration of the bowel loop was identified and correction was not complete with umbilical circulation.

**Table 3:** Statistics related to quantitative data of pregnant women and newborns for each surgical technique

Variable according to group		Median	Minimum	Maximum	Mann-Whitney test
Age at delivery, years					
	Silo	19	14	29	0.9240
	EXIT-like	19	15	33	
Gestational age at delivery, weeks					
	Silo	37.1	35.3	38.4	0.0713
	EXIT-like	35.5	35	36.4	
Weight at birth, g					
	Silo	2,270	1,700	3,480	0.5083
	EXIT-like	2,113	1,556	2,975	
Mechanical ventilation, days					
	Silo	22	2	26	0.0169
	EXIT-like	0.5	0	3	
Parenteral nutrition, days					
	Silo	45	26	55	0.0104
	EXIT-like	14.5	14	18	
Mixed nutrition, days					
	Silo	6	5	12	0.1262
	EXIT-like	10.5	7	22	
Enteral nutrition, days					
	Silo	53	31	60	0.0294
	EXIT-like	25.5	24	36	
Length of hospital stay, days					
	Silo	67	36	83	0.726
	EXIT-like	38.5	33	46	

We found no difference between the two groups, concerning maternal age at delivery (p=0.9240) and gestational age at delivery (p=0.0713). Examining the clinical data of the newborns, it was noted that weight at birth was statistically the same for both groups (p=0.5083). Nevertheless, in the postoperative period, the situation was different in the groups, especially if time in mechanical ventilation, parenteral nutrition and enteral nutrition were considered, since the EXITlike Group presented lower medians and more data homogeneity than the Silo Group, that is, there was low dispersion in the EXIT-like Group concerning these variables.

**Figure 4:**
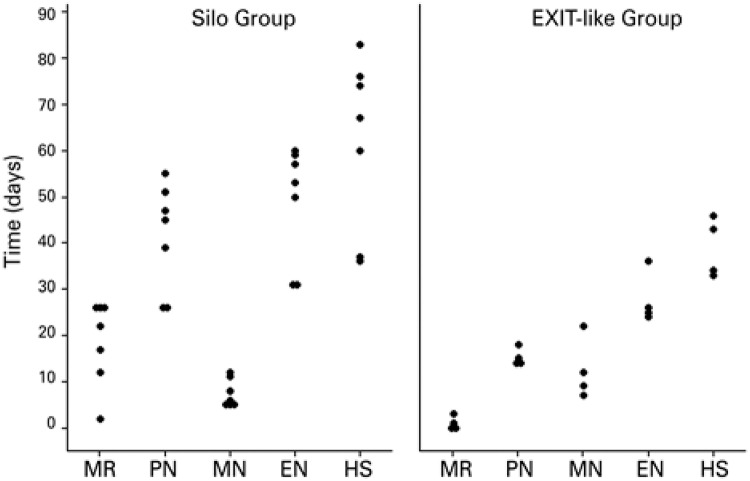
Graphic of individual values for the variables: mechanical ventilation duration (MR), parenteral nutrition (PN), mixed nutrition (MN), enteral nutrition (EN) and hospital stay (HS) for the two studied groups

Figure 4 shows the intra-group dispersion in a comparative way for all parameters considered. As for the mixed nutrition, statistics did not show difference between the groups, although medians suggested a more favorable result for the Silo Group, with shorter median time. That is possibly because of the high data dispersion considering this variable in the EXIT-like Group caused by nutrition duration (22 days) of one of the newborns. As for hospital stay, although median values were not numerically close (67 days for the Silo Group and 38.7 days for the EXIT-like Group), a statistically relevant difference was not verified in the groups, based on the Mann-Whitney test, possibly due to the high data dispersion of the Silo Group.

Neonatal sepsis was observed in only one case in the EXIT-like Group and in three cases in the Silo Group. Surgical reintervention was not necessary in the EXIT-like Group, while in the Silo Group, reintervention was necessary in three out of seven patients (42.9%).

## DISCUSSION

Gastroschisis is a frequent congenital defect that presents high neonatal morbidity rates and great costs for the health system. There is no consensus about how these malformation cases should be treated. Delivery mode, gestational age at delivery, best moment for surgical intervention and the selected technique vary among different health care centers, since scientific evidence is weak and does not consistently support any of the therapeutic strategies.^(^ ^12^ ^-^ ^17^ ^)^ We aimed to report our initial experience with a new ultrasonographic index and an innovative surgical technique to be applied in gastroschisis cases. The use of this index seems intuitive, since the difficulty in reducing the bowel loops and the risk of compartment syndrome are directly related to grade of edema, and it depends on the magnitude of the abdominal wall defect. We showed that when this index is favorable, the EXIT-like technique is applicable and seems safe, resulting in the same or better neonatal outcome than the classical approach we have been using in our unit.

In most centers, the classical approach is to perform primary closure in the first hours of life, with the newborn under general anesthesia, and in an operating room. Some health centers carry out primary repair in the neonatal intensive care unit, and use it as the first attempt of correction.^(^ ^12^ ^-^ ^17^ ^)^ The Silo is a synthetic bag designed to cover the gastroschisis and is fixed to the abdominal wall, normally the fascia. After placement, viscera are reduced one or two times a day until complete reduction, which usually occurs from 1 to 14 days after delivery.^(^ ^12^ ^-^ ^17^ ^)^

The two types of Silo most often used are the preformed (silicon) or the customized (PVC) devices. Preformed Silo may be placed in the delivery room, in the bassinet, with newborn under sedation. After finishing the reduction, abdominal wall closure is still necessary. This type of Silo aims at reducing morbidity, diminishing the necessity of neonatal anesthesia, at least in the first hours of life. The customized Silos are often “built” during surgical procedure, which requires newborn to be under general anesthesia.^(^ ^12^ ^-^ ^17^ ^)^ Silo placement and delayed closure seem to be especially indicated in cases with viscero-abdominal disproportion due to the risk of compartment syndrome. Despite lacking scientific relevance, many studies have been carried out comparing Silo to primary closure.^(^ ^15^ ^)^ There is a great variety of study designs, populations evaluated and results obtained. However, in the groups of newborns treated with Silo placement, mechanical ventilation was required for a shorter period,^(^ ^13^ ^-^ ^15^ ^)^ which can also be correlated with lower risk of hyponatremia and hypoalbunemia.^(^ ^19^ ^)^

The technique presented in our study is based on primary closure of the defect; however, without air inside bowel loops, that is, at “zero minute”. We believe this may be the main reason for the better postnatal parameters studied. The use of the classic EXIT technique would led to these results, though with the technique presented, this procedure may be quite simplified, avoiding maternal general anesthesia and the need of uterine relaxation during repair of the defect.^(^ ^20^ ^-^ ^26^ ^)^ In the novel technique described, only viscera reduction is performed with maternal circulation and, as the procedure is usually fast, the fetus may be totally exteriorized, as in a conventional cesarean section, considerably reducing the risks of uterine atony and the need of blood transfusion.

In the EXIT-like technique, fetal analgesia was given through maternal transplacental circulation using remifentanil, starting at least 15 minutes before fetal extraction. This drug was chosen because it easily crosses the placental barrier, and for its use in EXIT cases, when general anesthesia is not recommended.^(^ ^27^ ^,^ ^28^ ^)^ Sucrose solution is offered to the newborn to avoid air swallowing and for having a positive impact in pain management.^(^ ^29^ ^-^ ^31^ ^)^

The EXIT-like technique choice presumes cesarean section as the delivery mode. The option for a delayed elective preterm delivery at 35-37 gestational weeks is controversial, although relevant studies show that the average gestational age of spontaneous deliveries in cases of gastrochisis is very similar.^(^ ^11^ ^,^ ^12^ ^)^ Payne et al. showed prematurity was the only factor that demonstrated a better prognosis when compared to other parameters.^(^ ^32^ ^)^ We believe that the low risk of complications related to late prematurity, as well as a potential prevention of bowel loops damage due to prolonged exposure to the amniotic fluid, justify and support the anticipation of delivery.^(^ ^9^ ^,^ ^33^ ^)^

Although we have operated on only four cases so far, when analyzing the postoperative results, we have observed better outcome as compared to the delayed closure with Silo placement technique, which had been used before in our center. Table 4 shows the results of this study comparing primary repair and delayed closure with Silo placement and two other studies.^(^ ^12^ ^,^ ^13^ ^)^ We note that, except for the number of assisted ventilation days, which is greater in the primary closure group, all other parameters do not show statistically significant differences concerning primary closure *versus* delayed with Silo.^(^ ^12^ ^,^ ^13^ ^)^ Even taking into account that the result of the two groups was different with regard to the length of stay in days, and parenteral/enteral nutrition, this fact only reinforced the importance of the difference found in our group. We believe that the difference found between the two studies may be related to diverse neonatal protocols used in the two centers regarding the criteria for introducing nutrition and hospital discharge. In our cases, as the same protocol was applied in the Silo and in the EXIT-like groups, this may suggest this difference to be related to the new surgical technique described. In our experience, the EXIT-like significantly reduced the number of days of nutritional transition and hospital discharge. On the other hand, it is not yet possible to state that this reduction has occurred because of the surgical technique or late prematurity proposed in the EXIT-like Group *versus* term delivery, which was used in the Silo Group of this study.

**Table 4:** Comparison of results considering the EXIT-like, primary closure and Silo placement techniques

	EXIT-like n=4	Silo n=7	Charlesworth et al.^(^ ^13^ ^)^ n=67	Charlesworth et al.^(^ ^13^ ^)^ n=89	Stanger et al.^(^ ^12^ ^)^ n=300	Stanger et al.^(^ ^12^ ^)^ n=379
Characteristics	“Zero minute”	Silo	Primary closure	Silo	Primary closure	Silo
Days of mechanical ventilation, mean	1	18.71	5	3.5	6.79	7.04
Days of parenteral nutrition, mean	15.25	41.29	19	24	16.5	17.7
Das of exclusive enteral nutrition, mean	27.75	48.71	24	27	44.3	43.6
Days of hospital stay, mean	39	61.86	30	24	56.6	53.8

It is known that bowel loops exposure to the amniotic fluid during gestation may lead to wall thickening, changing peristalsis and mucosal absorption. For that reason, many ultrasonographic markers have been described in an attempt to demonstrate severity of gastroschisis, and they could predict an increase in neonatal morbidity and mortality.^(^ ^1^ ^,^ ^6^ ^,^ ^34^ ^,^ ^35^ ^)^ Although the clinical applicability of these parameters is controversial, there is no doubt that bowel loop lesions progress during gestation. They are probably caused by progressive exposure to the amniotic fluid and/or tissue ischemia associated to bowel dilation, leading to abdominal wall defect. Therefore, serial ultrasound control using SRI may be important since thickness increase and dilation of bowel loops are taken into account in the calculation. Nevertheless, other studies should be carried out for its clinical validation.

Since not all cases will be suitable for the EXIT-like procedure, the classical approach must remain available as an option, and it is very important to emphasize to patients that depending on surgical findings, the conversion to such approach will be mandatory. For this purpose, a second operating room should always be prepared in case the alternative procedure has to be aborted.

We believe our findings cannot yet establish the real impact of the SRI use in cases of gastroschisis in order to choose the correction surgical technique and the best moment of delivery. But the superiority of the EXIT-like procedure in our cases could encourage further research.

The small number of cases and the comparison of the study group and controls were important limitations of our study. However, at least in our center, this new approach represented a significant neonatal improvement and this may be a reality in other centers with scarce financial resources, especially in countries with large territorial distances.

## CONCLUSION

The use of EXIT-like procedure for gastroschisis correction was feasible in selected cases and may improve neonatal outcome by reducing stay at the neonatal intensive care unit time and neonatal morbidity. The successful application of this novel “zero minute” approach is an important first step for the introduction of a rather intuitive, but needed change in gastroschisis management, especially in countries with fewer financial resources. We believe our results ensure a randomized controlled trial to be undertaken.
